# Family 1 glycosyltransferases (GT1, UGTs) are subject to dilution-induced inactivation and low chemo stability toward their own acceptor substrates

**DOI:** 10.3389/fmolb.2022.909659

**Published:** 2022-07-22

**Authors:** David Teze, Gonzalo Nahuel Bidart, Ditte Hededam Welner

**Affiliations:** The Novo Nordisk Foundation Center for Biosustainability, Technical University of Denmark, Kongens Lyngby, Denmark

**Keywords:** glycosyltransferases, glycosylation, biotechnology, stability, polyphenols, GT1, UGT

## Abstract

Glycosylation reactions are essential but challenging from a conventional chemistry standpoint. Conversely, they are biotechnologically feasible as glycosyltransferases can transfer sugar to an acceptor with perfect regio- and stereo-selectivity, quantitative yields, in a single reaction and under mild conditions. Low stability is often alleged to be a limitation to the biotechnological application of glycosyltransferases. Here we show that these enzymes are not necessarily intrinsically unstable, but that they present both dilution-induced inactivation and low chemostability towards their own acceptor substrates, and that these two phenomena are synergistic. We assessed 18 distinct GT1 enzymes against three unrelated acceptors (apigenin, resveratrol, and scopoletin—respectively a flavone, a stilbene, and a coumarin), resulting in a total of 54 enzymes: substrate pairs. For each pair, we varied catalyst and acceptor concentrations to obtain 16 different reaction conditions. Fifteen of the assayed enzymes (83%) displayed both low chemostability against at least one of the assayed acceptors at submillimolar concentrations, and dilution-induced inactivation. Furthermore, sensitivity to reaction conditions seems to be related to the thermal stability of the enzymes, the three unaffected enzymes having melting temperatures above 55°C, whereas the full enzyme panel ranged from 37.4 to 61.7°C. These results are important for GT1 understanding and engineering, as well as for discovery efforts and biotechnological use.

## Introduction

**SCHEME 1:**
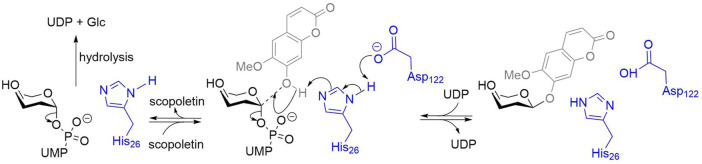
Scopoletin glucosylation by GT1s. Amino acids numbers from *Pt*UGT1 (Teze et al., 2021). The enzyme residues are represented in blue, the acceptor in grey, and the donor in black. A His activated by an Asp acts as a general base, increasing the nucleophilicity of the acceptor. The predominant reaction is the reversible glycosylation of scopoletin. The enzyme can also catalyze the irreversible hydrolysis of the donor uridine diphosphate glucose (UDP-Glc).

Glycosylation is one of the most common reactions in the biosphere, yet a particularly challenging one for conventional synthetic chemistry. Indeed, the need to control both regio- and stereo-selectivity leads to a succession of reactions, including protecting group manipulations and bond activations, resulting in low chemical yields, poor atom economy, and large amounts of waste. Conversely, enzymatic glycosylation occurs in a single reaction with unprotected sugars and acceptors and lends perfect control over stereoselectivity (Nidetzky et al., 2018). Provided with the appropriate enzyme, full control over regioselectivity, as well as quantitative chemical yields are also feasible. In Nature, glycosylation is primarily catalyzed by glycosyltransferases, enzymes that transfer a saccharide from an activated sugar donor to an acceptor molecule. These enzymes are organized in >100 distinct glycosyltransferase families in the CAZy database (Coutinho et al., 2003; Lombard et al., 2014), with all enzymes within a family sharing phylogeny, structural fold, and generally mechanism. The β-glycosylation of natural products is mainly achieved by enzymes from glycosyltransferase family 1 (GT1) (Louveau and Osbourn, 2019). These GT1s are inverting enzymes using α-nucleotide sugars as donors, most commonly UDP-sugars, and are thus also termed UGTs, for UDP-dependent glycosyltransferases (Ross et al., 2001). They catalyze the formation of *O*-, *N*-, *S*- or *C*-glycosidic bonds. *O*-glycosylations are the most common reactions and are usually promoted by a His-Asp catalytic dyad sharing a proton abstracted from the acceptor (Scheme 1) (Brazier-Hicks et al., 2007; Teze et al., 2021). The *N*- and *S*- mechanisms are slightly different (Teze et al., 2021), and the *C*-glycosylation mechanism is related but yet to be firmly established (Gutmann and Nidetzky, 2013; Putkaradze et al., 2021). GT1 enzymes are relatively promiscuous, being able to act on a variety of natural products (Offen et al., 2006; Chen et al., 2015; Zhang et al., 2022), and most GT1s are active against polyphenols (Yang et al., 2018).

GT1s have received considerable interest as tools for biotechnological glucosylation (Nidetzky et al., 2018; Vasudevan and Lee, 2020). Indeed, the possibility to use sucrose synthase for forming UDP-Glc from UDP and sucrose, and using lysates from the enzyme’s production as UDP providers, makes β-glucosylation an economically feasible process (Wang et al., 2012; Schmölzer et al., 2016; Liu and Nidetzky, 2021). However, their stability—a crucial industrial property—has only been scarcely characterized (Fujiwara et al., 2009; Gao et al., 2020). In a few recent cases (Petermeier et al., 2021; Bidart et al., 2022), we observed instability, seemingly not intrinsic but dependent on experimental conditions, particularly enzyme and acceptors concentrations. Indeed, a nonlinear behavior was observed upon enzyme dilution, particularly at acceptor substrate concentrations in the millimolar range (Petermeier et al., 2021; Bidart et al., 2022). In order to investigate how widespread this peculiar behavior is within GT1-catalyzed reactions, we analyzed the effect of 16 reaction conditions on end-point reaction yields from 18 distinct GT1 enzymes, each against three different polyphenol acceptors.

## Materials and methods

### Protein production, purification, and storage

Proteins are expressed in One Shot™ BL21 Star™ (DE3) *E. coli* cells (ThermoFisher Scientific, United States of America) cells transformed with pET28a + plasmids encoding the various enzymes with a hexahistidine tag and a TEV cleavage site in N-term (plasmids purchased from Genscript, United States). The native DNA sequences were retrieved from UniProt (Supplementary Table S1) and cloned at the multiple cloning site of the pET28a + plasmids. Protein expression is induced by the addition of 200 μM of isopropyl-β-d-galactopyranoside to cultures that had reached an optical density at 600 nm of 0.6 and continued for 16 h at 293 K. The cultures are then centrifuged, and the pellet is resuspended in 50 mM 4-(2-hydroxyethyl)-1-piperazineethanesulfonic acid (HEPES) pH 7, 300 mM NaCl, and 20 mM imidazole. The cell suspension is lysed in a homogenizer (French Press) Avestin Emulsiflex C5 (ATA Scientific Pty Ltd. Canada), centrifuged and the pellet is discarded. The supernatant is purified by nickel affinity chromatography (HisTrap^TM^ FF, GE Healthcare, Sweden) on an ÄKTA pure (GE Healthcare, Sweden). The fractions containing the purified GT1 are pooled, concentrated, buffer exchanged against 25 mM HEPES pH 7, 50 mM NaCl, and 1 mM dithiothreitol (DTT), then stored at 193 K after flash-freezing in 25 μl aliquots.

### Enzymatic reactions and yield determination

All reactions were performed in flat-bottom, low sorption 96-well microtiter plates, in the following conditions: 100 μL volume, no stirring, 20 h at 293 K. The reaction components were 10, 20, 40, or 80 mg/L (circa 0.15–1.2 μM) enzyme; 500 μM UDP-Glc; 50, 100, 200, or 400 μM aglycon; and 25 mM HEPES pH 7. After 20 h, reactions were diluted 25-fold in milli-Q water (10 + 240 μl) and analyzed by reverse-phase chromatography. Acceptor consumption was monitored according to a standard curve, using an Ultimate 3,000 Series apparatus (Thermo Scientific) and an Eclipse Plus C18 3.5 µm 100 × 4.6 mm analytical column (Agilent). Milli-Q water containing 0.1% formic acid and acetonitrile were used as mobile phases A and B, respectively. Monitoring and data handling was operated using the Chromeleon software (Thermo Scientific). A combination of isocratic, immediate ramp, and gradients at a flow rate of 1 ml/min was used for the analytes separation: 0–0.5 min, 2% B; 0.5–1.5 min, 35% B; 1.5–3 min, 35–80% B; 3–4.2 min, 98% B; 4.2–5 min, 2% B. Apigenin and scopoletin were monitored at 340 nm, resveratrol at 300 nm. Data points for which acceptor consumption did not match product appearance were discarded.

### Enzymatic rates measurement

Reactions were performed in flat-bottom, low sorption 96-well microtiter plates, in the following conditions: 100 μl volume, no stirring, 293 K. The reaction components were 20 or 80 mg/L enzyme; 500 μM UDP-Glc; 50 or 400 μM apigenin; and 50 mM HEPES pH 7. UDP-Glc is added last to start the reaction. After 0.5, 2.5, 4.5, 6.5, 8.5, and 10.5 min, 10 μL aliquots were quenched by a 25-fold dilution of 0.1% acetic acid (10 + 240 μl) and analyzed by reverse-phase chromatography as reported earlier.

### Differential scanning fluorimetry

Melting temperatures (*T*_m_) of the different UGTs were measured by DSF using the Protein Thermal Shift Dye Kit (ThermoFisher Scientific) and a qPCR QuantStudio5 machine. Dye solution (1,000×) and acceptors (resveratrol, scopoletin, apigenin, quercetin, pinoresinol, silibinin, xanthotoxol, genistein, and 3,4-dichlorophenol) were diluted in 0.8 equivalents NaOH in H_2_O milliQ (e.g., 1 mM acceptor in 800 μM NaOH). 10 μL of dye/acceptor solution 2x was mixed with 10 μL of 0.8 mg/ml enzyme samples in 2 × buffer (100 mM HEPES pH7) and pipetted into a qPCR 96-wells plate. Final conditions were thus HEPES pH7 50 mM, 0.4 mg/ml enzyme, acceptor either 0, 400 μM (polyphenols) or 750 μM (3,4-dichlorophenol). The plate was centrifuged for 30 s at 1,000 rpm and transferred to the qPCR machine. The protocol initiates with 2 min incubation at 298 K, followed by a temperature increase of 0.05 K s^−1^ up to 372 K, and a final incubation of 2 min at 372 K. Measurements were carried out in triplicate. Raw data were analyzed with Protein Thermal Shift™ Software v1.x.

### Native PAGE

Aliquots of purified *Fi*88A10 were diluted with Novex™Tris-Glycine Native Sample Buffer to final 0.25 μg/μl; 0.5 μg/μl; 1 μg/μl; 2 μg/μl and 2.5 μg/μl 10 μl of each sample (16 μl for the sample at 2.5 μg/μl, 40 μg final) were loaded and run on a Novex™ WedgeWell™ 10%, Tris-Glycine, 1.00 mm, Mini Protein Gel (ThermoFisher Scientific, United States) for 60 min at 215 V. The gel was stained with InstantBlue^®^ (Abcam, United Kingdom) for 15 min and rinsed with water before acquiring the picture.

## Results

The 18 GT1 enzymes have 24–40% pairwise identity after multiple sequence alignment *via* Clustal Omega (Sievers and Higgins, 2014). Nine of these enzymes have been previously described in the literature: *Pt*UGT1 (Teze et al., 2021), *Zm*UGT708A6 (Ferreyra et al., 2013), *Zm*UGT706F8 (Bidart et al., 2022), the GT1s from *Arabidopsis thaliana* (*At*UGT72E2, *At*71C1, *At*71D1) (Yang et al., 2018), RhGT1 (Wang et al., 2013), Gm88E3 (Liu and Nidetzky, 2021), and *Mt*UGT78G1 (Modolo et al., 2007). Among the nine GT1 enzymes that were not previously described, five already had designated names (*Zm*71B1, *Os*88C1, *Lc*72B10, *Fi*88A10, and *Fe*88J1), and the remaining four are named according to the UGT naming convention (Mackenzie et al., 2005) preceded by two letters referring to genus and species (e.g., *Zm*UGT88C10). UniProt accession numbers and melting temperatures (*T*_m_) of the 18 GT1 enzymes are provided in Supplementary Table S1.

**FIGURE 1:**
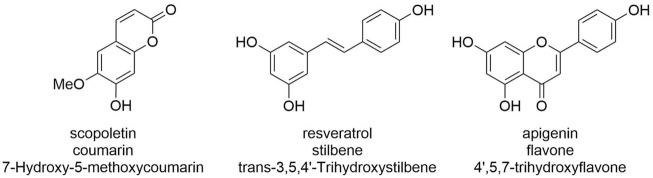
Acceptors assessed in this study. Note that while only a single glucosylation product is possible for scopoletin, several could be—and are—formed by GT1 enzymes for resveratrol and apigenin. Given that different products or product mixtures are observed for the various enzymatic: substrate pairs, the displayed analytical yields relate to acceptor consumption and are cross-validated by analyzing the sum of the peak areas observed for products.

**FIGURE 2:**
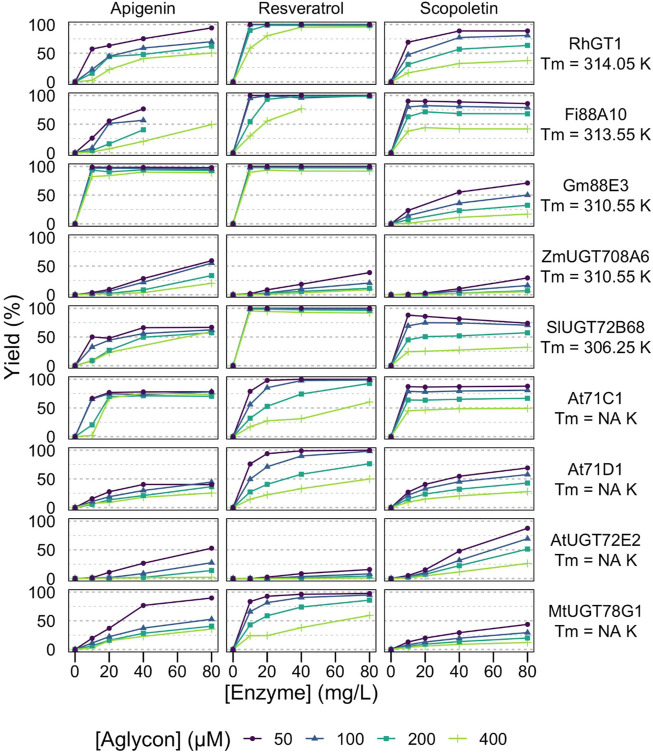
Effect of reaction conditions on glycosylation yields from low-*T*_m_ enzymes. Analytic yields of acceptor conversion are plotted against enzyme concentration, from 10 to 80 mg/L (circa 0.15–1.2 μM). HEPES pH 7, aglycon concentration range 50–400 μM, UDP-Glc 500 μM, 20 h at 293 K, without stirring in 100 μl volume. The 9 GT1s with the lowest *T*_m_ or no measured *T*_m_ are displayed. NA = Not Available, as the fluorescence as a function of the temperature did not reflect a classical behavior melting temperature.

**FIGURE 3:**
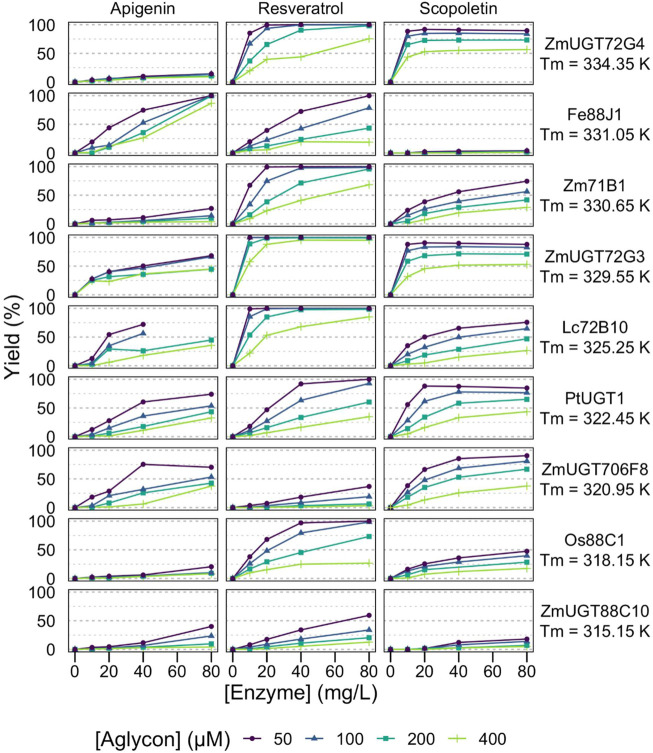
Effect of reaction conditions on glycosylation yields from high-*T*_m_ enzymes. Analytic yields of acceptor conversion are plotted against enzyme concentration, from 10 to 80 mg/L (circa 0.15–1.2 μM). HEPES pH 7, aglycon concentration range 50–400 μM, UDP-Glc 500 μM, 20 h at 293 K, without stirring in 100 μL volume.

These 18 GT1 enzymes are described here for their activity against three acceptors (Figure 1), representing different classes of polyphenols of biotechnological interest, that is, coumarins (scopoletin), stilbenes (*trans*-resveratrol), and flavones (apigenin). Interestingly, we found that each of the 18 enzymes is active against each of the chosen acceptors, and in most cases (44/54), analytical yields of glycosylation >50% are reached. Greater than 90% of yields are obtained for at least one condition in about half of the enzyme-acceptor pairs (Figures 2, 3).

While most of the curves display the classical dependency on enzyme concentration of a reaction catalyzed by enzymes with low total turnover numbers—that is, a linear or sublinear increase in the product as a function of enzyme concentration—half of the enzyme: substrate pairs (27/54) display dilution-induced inactivation behavior with a superlinear dependency on enzyme concentration (Figures 2, 3). At low enzyme concentrations (e.g., 10 mg/L, circa 150 nM), the no-to-little reaction is observed, yet doubling the enzyme concentration far more than double the observed yields. The full dataset of yields as a function of enzyme and acceptor concentrations is available in the supplementary material (Supplementary Table S2).

Importantly, this behavior is also related to acceptor concentration, being more prevalent at 400 μM than at 50 μM. It is particularly pronounced with apigenin, for example, *Fe*88J1, *Pt*UGT1, *Zm*UGT88C10, *At*UGT72E2, *At*71C1, *Rh*GT1, and *Zm*UGT708A6 (Figures 2, 3). It is also observed with resveratrol (e.g., *Zm*UGT708A6 or *Zm*UGT706F8) and scopoletin (e.g., *Zm*UGT88C10 or *At*UGT72E2). Interestingly, while glycosylation of apigenin and resveratrol regularly (19/36) reaches full conversion of the acceptors, the glucosylation of scopoletin results in an equilibrium (Scheme 1), with a maximum yield depending on the acceptor concentration. At the highest acceptor concentration, nearing donor and acceptor equimolarity (500 and 400 μM, respectively), the maximal yields observed are around 50% (Figures 2, 3). This allows for the observation of hydrolysis in 5/18 GT1 enzymes in our dataset, being particularly pronounced for SlUGT72B68. Indeed, while the formation of scopoletin-glucoside from UDP-Glc and the formation of UDP-Glc from scopoletin are in equilibrium, the hydrolysis of UDP-Glc by the enzyme is irreversible (Scheme 1). There seems to be a weak relationship between the intrinsic stability of the enzyme, represented by its melting temperature (*T*_m_), as the three enzymes seemingly unaffected by the conditions were the relatively stable *Zm*UGT72G3 (*T*_m_ = 56.4 ± 0.1⁰C/*T*_m_ = 329.6 ± 0.1 K), *Zm*UGT72G4 (*T*_m_ = 61.2 ± 0.4⁰C/*T*_m_ = 334.4 ± 0.4 K), and *At*71D1 (ND). Conversely, *Zm*UGT708A6 (*T*_m_ = 37.4 ± 0.1⁰C/*T*_m_ = 310.6 ± 0.1 K) and *Zm*UGT88C10 (*T*_m_ = 42 ± 0.3⁰C/*T*_m_ = 315.2 ± 0.3 K) were most affected by conditions. Considering ∼0.008 kJ/mol/residue (Rees and Robertson, 2001), and an average length of GT1 enzymes of *c.* 500 residues, a Δ*T*_m_ of 1 K roughly equates to stabilization of 1 kcal/mol, thus between the most and least stable enzymes in our dataset a difference as large as 25 kcal/mol is observed. Enzyme-substrate interactions are generally thought to be stabilizing, which is the rationale behind the use of differential scanning fluorimetry as a basis for identifying enzyme-substrate pairs (Niesen et al., 2007). We assessed whether polyphenol acceptors modified the *T*_m_ of our proteins, and did not observe a significant change in either direction (Supplementary Figure S2). *Zm*UGT708A6, which displays chemostability issues in presence of all three acceptors, would even appear to present slightly higher *T*_m_ in presence of resveratrol and apigenin (Supplementary Figure S1).

## Discussion

In this article, we demonstrate the widespread yet not widely reported phenomena of dilution-induced inactivation and low chemostability towards their own acceptors of GT1 enzymes. These effects are important and can introduce biases in both the kinetic study and discovery efforts for GT1 enzymes. The latter is of particular importance since one of the major obstacles to a wider biotechnological application of glycosyltransferases is the characterization of their acceptor scope. While one might be enticed to assess acceptors at high concentrations to detect catalysts with low affinity (high *K*_S_), or at low enzyme concentrations to be cost-efficient, our results demonstrate that this would result in a significant number of false negatives. While we report the effect, we do not offer a mechanistic explanation. Protein destabilization by small molecules generally occurs at much higher concentrations than the effects reported here at submillimolar concentrations (Singh et al., 2011). Molecular crowding, occasionally invoked to rationalize dilution-induced inactivation, occurs at much higher concentrations (Miklos et al., 2011; Wang et al., 2012; Cohen and Pielak, 2017). Conversely, the enzyme’s adsorption onto equipment (vessel, glassware, tips, etc.) is a concern for trace concentrations or up to the nanomolar range, several orders of magnitude lower than our data and therefore not likely to account for our observations. Furthermore, GT1 enzymes are monomeric, clearly demonstrated by size exclusion chromatography and several crystallographic structures (Wetterhorn et al., 2016). Furthermore, we performed a native PAGE analysis of *Fi*88A10 at different concentrations, up to 2.5 μg/μl (>300-fold higher concentration than the highest concentration used in the enzymatic assays). Even at high concentration, *Fi*88A10 appeared monomeric, ruling out dilution-induced oligomerization disruption as an explanation (Supplementary Figure S2). The effects seemed to affect enzymes presenting either high (e.g., *At*71C1 on apigenin) or low (e.g., *Zm*UGT708A6 on apigenin) specificity. To assess whether initial rates were affected, we determined these rates for three enzymes (*Fi*88A10, *At*71C1, and *Zm*UGT708A6) at defined conditions (20 or 80 mg/L enzymes, 50 or 400 μM apigenin). We observed a strong effect on the chemo instability, as no rates/reaction could be observed within 10 min for *Fi*88A10 and *Zm*UGT708A6 at 400 μM acceptor concentration, particularly compared to the reasonable rates (8–60 per min) observed at 50 μM apigenin (Supplementary Table S3). Conversely, the initial rates of *At*71C1 in presence of 50 μM apigenin could not be determined, as >30% of the acceptor was converted within 0.5 min even at 20 mg/L enzymes.

Here, the synergistic effect with the chemostability at moderately high acceptor concentrations, together with the fact that each enzyme presents various behaviors depending on the acceptor, indicates that specific phenomena related to GT1 enzymes are behind our observations. Conceivably, their relatively large, solvent-exposed hydrophobic acceptor site (Brazier-Hicks et al., 2007; Teze et al., 2021) could be involved. Appreciation of these effects is important both for mechanistic and kinetic studies of GT1 enzymes, their biotechnological use (scale-up to high acceptor concentrations), as well as for discovery efforts.

## Data Availability

The original contributions presented in the study are included in the article/Supplementary Material, further inquiries can be directed to the corresponding authors.
