# Supplementary material for: Family 1 glycosyltransferases (GT1, UGTs) are subject to dilution-induced inactivation and low chemo stability toward their own acceptor substrates
Source: Front Mol Biosci. 2022 Jul 22;9:909659. doi: 10.3389/fmolb.2022.909659 (PMC9354691; doi:10.3389/fmolb.2022.909659)
Supplement: Supplementary file 1 [file DataSheet1.PDF]

Supplementary Material

Supplementary Table 1. Uniprot accession numbers and melting temperatures of the enzymes used in the dataset. Standard deviations given for the enzymes' T_m are the result of triplicates.

Enzyme name	Organism of origin	Uniprot ID	T_m (°C)	Prior references
*Zm*_UGT72G3	*Zea mais*	B6SRY5	56.4±0.1	
*Zm*_UGT72G4	*Zea mais*	B4F9H1	61.2±0.4	
*Zm*_UGT708A6	*Zea mais*	A0A096SR	37.4±0.1	(Ferreyra *et al.*, 2013)
*Zm*_UGT88C10	*Zea mais*	C0HFA0	42±0.3	
*Zm*_UGT706F8	*Zea mais*	B4FG90	47.8±0.5	(Bidart *et al.*, 2022)
*Zm*_71B1	*Zea mais*	A0A1D6ICF	57.5±0.2	
*Sl*_UGT72B68	*Solanum lycopersicum*	D7S016	33.1±0.2	
*Rh*_GT1	*Rosa hybrid cultivar*	Q4R1I9	40.9±0.2	(Wang *et al.*, 2013)
*Os*_88C1	*Oryza sativa (rice)*	Q8LJ11	45±0.6	
*Lc*_72B10	*Lycium chinense*	B6EWZ3	52.1±0.2	
*Gm*_88E3	*Glycine max (soybean)*	A6BM07	37.4±0.2	(Liu and Nidetzky, 2021)
*Fi*_88A10	*Forsythia x intermedia*	D2KY82	40.4±0.4	
*Fe*_88J1	*Fagopyrum esculentum*	A0A0A1H7	57.9±0.3	
*At*_71D1	*Arabidopsis thaliana*	O82383	NA	(Yang *et al.*, 2018)
*At*_71C1	*Arabidopsis thaliana*	O82381	NA	(Yang *et al.*, 2018)
*Pt*UGT1	*Polygonum tinctorium*	A0A2R2JFJ4	49.3±0.2	(Teze *et al.*, 2021)
*At*_UGT72E2	*Arabidopsis thaliana*	Q9LVR1	NA	(Yang *et al.*, 2018)
*Mt*_UGT78G1	*Medicago truncatula*	A6XNC6	NA	(Modolo *et al.*, 2007)

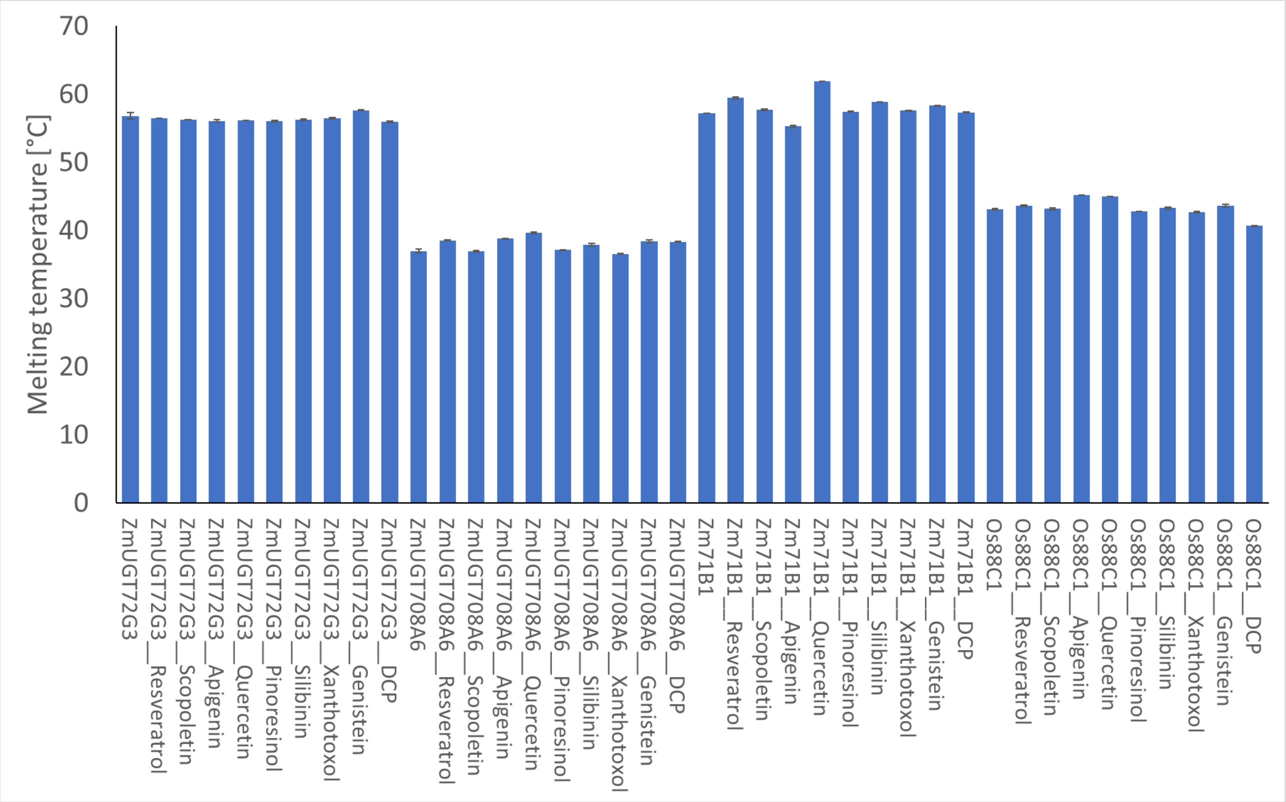

Supplementary Figure 1. Effect of candidate glycosyl acceptors on the melting point of four GT1 enzymes. Melting temperatures (T_m) were measured by differential scanning fluorimetry using the Protein Thermal Shift Dye Kit (ThermoFisher Scientific). Enzymes were assessed at 0.4 g/L in 50 mM HEPES buffer pH7, in presence of 0, 400 µM (polyphenols) or 750 µM (DCP, 3,4-dichlorophenol). Error bars represent the standard deviation of triplicates.

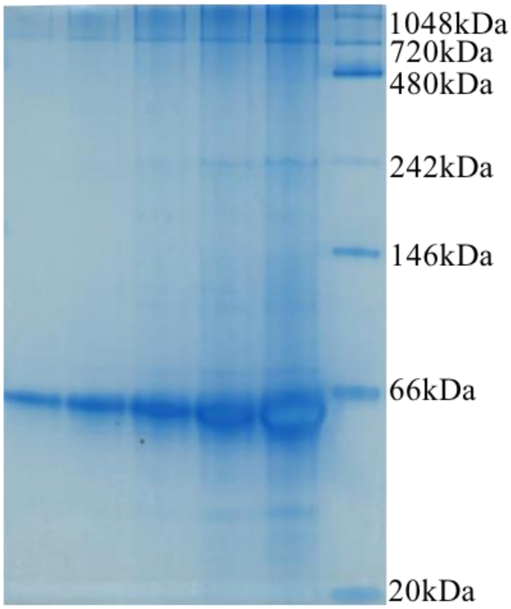

Supplementary Figure 2. *Fi*88A10 is monomeric even at high concentrations. A native PAGE gel analysis of Fi88A10 at concentrations of 0.25 μg/μL; 0.5 μg/μL; 1 μg/μL; 2 μg/μL and 2.5 μg/μL (left to right) show that the enzyme stays monomeric even at high protein concentration.

Supplementary Table 2. Analytical yields of glucosylation according to the GT1 enzyme, its concentration, the glucosylation acceptor, and its concentration.

Enzyme	Aglycon	[Enzyme] (mg/L)	[Aglycon] (µM)	Yield (%)
ZmUGT72G3	Apigenin	10	400	24.3
ZmUGT72G3	Apigenin	10	200	25.1
ZmUGT72G3	Apigenin	10	100	27.4
ZmUGT72G3	Apigenin	10	50	27.6
ZmUGT72G3	Apigenin	20	400	23.2
ZmUGT72G3	Apigenin	20	200	31.6
ZmUGT72G3	Apigenin	20	100	40.2
ZmUGT72G3	Apigenin	20	50	40.2
ZmUGT72G3	Apigenin	40	400	36.4
ZmUGT72G3	Apigenin	40	200	35.6
ZmUGT72G3	Apigenin	40	100	46.7
ZmUGT72G3	Apigenin	40	50	50.3
ZmUGT72G3	Apigenin	80	400	44.9
ZmUGT72G3	Apigenin	80	200	44.7
ZmUGT72G3	Apigenin	80	100	66.3
ZmUGT72G3	Apigenin	80	50	68.1
ZmUGT72G4	Apigenin	10	400	2.2
ZmUGT72G4	Apigenin	10	200	2
ZmUGT72G4	Apigenin	10	100	3.4
ZmUGT72G4	Apigenin	10	50	3.5
ZmUGT72G4	Apigenin	20	400	3.1
ZmUGT72G4	Apigenin	20	200	4.5
ZmUGT72G4	Apigenin	20	100	6.3
ZmUGT72G4	Apigenin	20	50	5.8
ZmUGT72G4	Apigenin	40	400	7
ZmUGT72G4	Apigenin	40	200	7.2
ZmUGT72G4	Apigenin	40	100	7.7
ZmUGT72G4	Apigenin	40	50	9.9
ZmUGT72G4	Apigenin	80	400	9.3
ZmUGT72G4	Apigenin	80	200	10.6
ZmUGT72G4	Apigenin	80	100	14
ZmUGT72G4	Apigenin	80	50	14.3
ZmUGT708A6	Apigenin	10	400	1.3
ZmUGT708A6	Apigenin	10	200	1.6
ZmUGT708A6	Apigenin	10	100	2
ZmUGT708A6	Apigenin	10	50	3.7
ZmUGT708A6	Apigenin	20	400	1.5
ZmUGT708A6	Apigenin	20	200	2.4
ZmUGT708A6	Apigenin	20	100	6.2
ZmUGT708A6	Apigenin	20	50	9.3

ZmUGT708A6	Apigenin	40	400	3.6
ZmUGT708A6	Apigenin	40	200	8.4
ZmUGT708A6	Apigenin	40	100	21.7
ZmUGT708A6	Apigenin	40	50	28.2
ZmUGT708A6	Apigenin	80	400	20
ZmUGT708A6	Apigenin	80	200	33.4
ZmUGT708A6	Apigenin	80	100	55.2
ZmUGT708A6	Apigenin	80	50	59.3
ZmUGT88C10	Apigenin	10	400	0.8
ZmUGT88C10	Apigenin	10	200	1
ZmUGT88C10	Apigenin	10	100	1.2
ZmUGT88C10	Apigenin	10	50	3.5
ZmUGT88C10	Apigenin	20	400	1.2
ZmUGT88C10	Apigenin	20	200	1.7
ZmUGT88C10	Apigenin	20	100	2.5
ZmUGT88C10	Apigenin	20	50	4.7
ZmUGT88C10	Apigenin	40	400	3.1
ZmUGT88C10	Apigenin	40	200	3.8
ZmUGT88C10	Apigenin	40	100	6.9
ZmUGT88C10	Apigenin	40	50	11.6
ZmUGT88C10	Apigenin	80	400	4.2
ZmUGT88C10	Apigenin	80	200	9.7
ZmUGT88C10	Apigenin	80	100	23.5
ZmUGT88C10	Apigenin	80	50	40
ZmUGT706F8	Apigenin	10	400	1
ZmUGT706F8	Apigenin	10	200	0.7
ZmUGT706F8	Apigenin	10	100	3.2
ZmUGT706F8	Apigenin	10	50	18.3
ZmUGT706F8	Apigenin	20	400	1.1
ZmUGT706F8	Apigenin	20	200	8
ZmUGT706F8	Apigenin	20	100	20.8
ZmUGT706F8	Apigenin	20	50	28.7
ZmUGT706F8	Apigenin	40	400	6.2
ZmUGT706F8	Apigenin	40	200	25.6
ZmUGT706F8	Apigenin	40	100	31.8
ZmUGT706F8	Apigenin	40	50	75.4
ZmUGT706F8	Apigenin	80	400	37.4
ZmUGT706F8	Apigenin	80	200	42.6
ZmUGT706F8	Apigenin	80	100	53.3
ZmUGT706F8	Apigenin	80	50	70.3
Zm71B1	Apigenin	10	400	1.4
Zm71B1	Apigenin	10	200	1.3
Zm71B1	Apigenin	10	100	1.5
Zm71B1	Apigenin	10	50	6.2

Zm71B1	Apigenin	20	400	1.6
Zm71B1	Apigenin	20	200	2.3
Zm71B1	Apigenin	20	100	2.7
Zm71B1	Apigenin	20	50	7
Zm71B1	Apigenin	40	400	2.9
Zm71B1	Apigenin	40	200	4.1
Zm71B1	Apigenin	40	100	5.6
Zm71B1	Apigenin	40	50	10.9
Zm71B1	Apigenin	80	400	3.9
Zm71B1	Apigenin	80	200	9.9
Zm71B1	Apigenin	80	100	14.3
Zm71B1	Apigenin	80	50	26.9
SlUGT72B68	Apigenin	10	400	8.1
SlUGT72B68	Apigenin	10	200	8.7
SlUGT72B68	Apigenin	10	100	32.7
SlUGT72B68	Apigenin	10	50	50.1
SlUGT72B68	Apigenin	20	400	23.1
SlUGT72B68	Apigenin	20	200	26.8
SlUGT72B68	Apigenin	20	100	44.6
SlUGT72B68	Apigenin	20	50	47.9
SlUGT72B68	Apigenin	40	400	NA
SlUGT72B68	Apigenin	40	200	49.6
SlUGT72B68	Apigenin	40	100	56
SlUGT72B68	Apigenin	40	50	66.2
SlUGT72B68	Apigenin	80	400	59.4
SlUGT72B68	Apigenin	80	200	57.5
SlUGT72B68	Apigenin	80	100	62.5
SlUGT72B68	Apigenin	80	50	66.9
RhGT1	Apigenin	10	400	2.9
RhGT1	Apigenin	10	200	15.11
RhGT1	Apigenin	10	100	21.6
RhGT1	Apigenin	10	50	57.4
RhGT1	Apigenin	20	400	21.5
RhGT1	Apigenin	20	200	44.1
RhGT1	Apigenin	20	100	44.6
RhGT1	Apigenin	20	50	63.3
RhGT1	Apigenin	40	400	40.8
RhGT1	Apigenin	40	200	47.9
RhGT1	Apigenin	40	100	59
RhGT1	Apigenin	40	50	75.3
RhGT1	Apigenin	80	400	50.5
RhGT1	Apigenin	80	200	62.1
RhGT1	Apigenin	80	100	70.1

RhGT1	Apigenin	80	50	94.1
Os88C1	Apigenin	10	400	0.9
Os88C1	Apigenin	10	200	0.9
Os88C1	Apigenin	10	100	1.9
Os88C1	Apigenin	10	50	2.3
Os88C1	Apigenin	20	400	0.8
Os88C1	Apigenin	20	200	1
Os88C1	Apigenin	20	100	2.6
Os88C1	Apigenin	20	50	4
Os88C1	Apigenin	40	400	3.6
Os88C1	Apigenin	40	200	3.9
Os88C1	Apigenin	40	100	4.4
Os88C1	Apigenin	40	50	6.1
Os88C1	Apigenin	80	400	8.2
Os88C1	Apigenin	80	200	9
Os88C1	Apigenin	80	100	10.1
Os88C1	Apigenin	80	50	20.5
Lc72B10	Apigenin	10	400	0.2
Lc72B10	Apigenin	10	200	2.5
Lc72B10	Apigenin	10	100	4.2
Lc72B10	Apigenin	10	50	12.6
Lc72B10	Apigenin	20	400	6
Lc72B10	Apigenin	20	200	29.2
Lc72B10	Apigenin	20	100	34.8
Lc72B10	Apigenin	20	50	54.3
Lc72B10	Apigenin	40	400	18
Lc72B10	Apigenin	40	200	26
Lc72B10	Apigenin	40	100	56.4
Lc72B10	Apigenin	40	50	72.1
Lc72B10	Apigenin	80	400	35.6
Lc72B10	Apigenin	80	200	44.8
Lc72B10	Apigenin	80	100	NA
Lc72B10	Apigenin	80	50	NA
Gm88E3	Apigenin	10	400	82.3
Gm88E3	Apigenin	10	200	93.6
Gm88E3	Apigenin	10	100	97.1
Gm88E3	Apigenin	10	50	98.9
Gm88E3	Apigenin	20	400	84
Gm88E3	Apigenin	20	200	90.5
Gm88E3	Apigenin	20	100	97
Gm88E3	Apigenin	20	50	98
Gm88E3	Apigenin	40	400	90.1
Gm88E3	Apigenin	40	200	94.1
Gm88E3	Apigenin	40	100	96.6

Gm88E3	Apigenin	40	50	98.4
Gm88E3	Apigenin	80	400	89
Gm88E3	Apigenin	80	200	92.8
Gm88E3	Apigenin	80	100	95.2
Gm88E3	Apigenin	80	50	97.9
Fi88A10	Apigenin	10	400	1.5
Fi88A10	Apigenin	10	200	4.1
Fi88A10	Apigenin	10	100	8.4
Fi88A10	Apigenin	10	50	25.6
Fi88A10	Apigenin	20	400	7
Fi88A10	Apigenin	20	200	15.7
Fi88A10	Apigenin	20	100	51.3
Fi88A10	Apigenin	20	50	55.6
Fi88A10	Apigenin	40	400	20.1
Fi88A10	Apigenin	40	200	40.2
Fi88A10	Apigenin	40	100	56.7
Fi88A10	Apigenin	40	50	76.6
Fi88A10	Apigenin	80	400	49.3
Fi88A10	Apigenin	80	200	NA
Fi88A10	Apigenin	80	100	NA
Fi88A10	Apigenin	80	50	NA
Fe88J1	Apigenin	10	400	1
Fe88J1	Apigenin	10	200	0.1
Fe88J1	Apigenin	10	100	9
Fe88J1	Apigenin	10	50	19.2
Fe88J1	Apigenin	20	400	11.8
Fe88J1	Apigenin	20	200	9.9
Fe88J1	Apigenin	20	100	13.6
Fe88J1	Apigenin	20	50	43.9
Fe88J1	Apigenin	40	400	26.3
Fe88J1	Apigenin	40	200	35.5
Fe88J1	Apigenin	40	100	52.8
Fe88J1	Apigenin	40	50	74.6
Fe88J1	Apigenin	80	400	86.7
Fe88J1	Apigenin	80	200	99.5
Fe88J1	Apigenin	80	100	99.9
Fe88J1	Apigenin	80	50	99.9
At71D1	Apigenin	10	400	7.5
At71D1	Apigenin	10	200	5.6
At71D1	Apigenin	10	100	10.6
At71D1	Apigenin	10	50	15.5
At71D1	Apigenin	20	400	9.1
At71D1	Apigenin	20	200	13.5

At71D1	Apigenin	20	100	18.9
At71D1	Apigenin	20	50	27.6
At71D1	Apigenin	40	400	17.9
At71D1	Apigenin	40	200	20.9
At71D1	Apigenin	40	100	29.8
At71D1	Apigenin	40	50	40.3
At71D1	Apigenin	80	400	25.4
At71D1	Apigenin	80	200	36.4
At71D1	Apigenin	80	100	44.4
At71D1	Apigenin	80	50	40
At71C1	Apigenin	10	400	2.1
At71C1	Apigenin	10	200	20.5
At71C1	Apigenin	10	100	65.5
At71C1	Apigenin	10	50	66.6
At71C1	Apigenin	20	400	67.2
At71C1	Apigenin	20	200	69.8
At71C1	Apigenin	20	100	74.9
At71C1	Apigenin	20	50	76.9
At71C1	Apigenin	40	400	75.5
At71C1	Apigenin	40	200	71.2
At71C1	Apigenin	40	100	73.3
At71C1	Apigenin	40	50	78
At71C1	Apigenin	80	400	73.6
At71C1	Apigenin	80	200	70.4
At71C1	Apigenin	80	100	78.1
At71C1	Apigenin	80	50	78.2
PtUGT1	Apigenin	10	400	1.2
PtUGT1	Apigenin	10	200	1.2
PtUGT1	Apigenin	10	100	3.5
PtUGT1	Apigenin	10	50	12.4
PtUGT1	Apigenin	20	400	1.2
PtUGT1	Apigenin	20	200	6.1
PtUGT1	Apigenin	20	100	15.2
PtUGT1	Apigenin	20	50	27.8
PtUGT1	Apigenin	40	400	11.1
PtUGT1	Apigenin	40	200	17.7
PtUGT1	Apigenin	40	100	35.9
PtUGT1	Apigenin	40	50	60.5
PtUGT1	Apigenin	80	400	32.6
PtUGT1	Apigenin	80	200	43.4
PtUGT1	Apigenin	80	100	53.7
PtUGT1	Apigenin	80	50	74
AtUGT72E2	Apigenin	10	400	0.3
AtUGT72E2	Apigenin	10	200	0.8

AtUGT72E2	Apigenin	10	100	1
AtUGT72E2	Apigenin	10	50	1.4
AtUGT72E2	Apigenin	20	400	0.6
AtUGT72E2	Apigenin	20	200	1
AtUGT72E2	Apigenin	20	100	1.6
AtUGT72E2	Apigenin	20	50	10.8
AtUGT72E2	Apigenin	40	400	1.6
AtUGT72E2	Apigenin	40	200	1.9
AtUGT72E2	Apigenin	40	100	8.7
AtUGT72E2	Apigenin	40	50	26.6
AtUGT72E2	Apigenin	80	400	2.2
AtUGT72E2	Apigenin	80	200	14
AtUGT72E2	Apigenin	80	100	27.6
AtUGT72E2	Apigenin	80	50	52.8
MtUGT78G1	Apigenin	10	400	2.7
MtUGT78G1	Apigenin	10	200	6.5
MtUGT78G1	Apigenin	10	100	11.3
MtUGT78G1	Apigenin	10	50	19.6
MtUGT78G1	Apigenin	20	400	15.2
MtUGT78G1	Apigenin	20	200	16
MtUGT78G1	Apigenin	20	100	22.3
MtUGT78G1	Apigenin	20	50	36.9
MtUGT78G1	Apigenin	40	400	22.8
MtUGT78G1	Apigenin	40	200	28.2
MtUGT78G1	Apigenin	40	100	37.2
MtUGT78G1	Apigenin	40	50	76.7
MtUGT78G1	Apigenin	80	400	35.6
MtUGT78G1	Apigenin	80	200	40.6
MtUGT78G1	Apigenin	80	100	52.7
MtUGT78G1	Apigenin	80	50	89.7
ZmUGT72G3	Resveratrol	10	400	57.7
ZmUGT72G3	Resveratrol	10	200	88.8
ZmUGT72G3	Resveratrol	10	100	99.8
ZmUGT72G3	Resveratrol	10	50	100
ZmUGT72G3	Resveratrol	20	400	88
ZmUGT72G3	Resveratrol	20	200	98.7
ZmUGT72G3	Resveratrol	20	100	99.8
ZmUGT72G3	Resveratrol	20	50	100
ZmUGT72G3	Resveratrol	40	400	95.3
ZmUGT72G3	Resveratrol	40	200	99.3
ZmUGT72G3	Resveratrol	40	100	99.8
ZmUGT72G3	Resveratrol	40	50	100
ZmUGT72G3	Resveratrol	80	400	95.1

ZmUGT72G3	Resveratrol	80	200	99.1
ZmUGT72G3	Resveratrol	80	100	99.8
ZmUGT72G3	Resveratrol	80	50	100
ZmUGT72G4	Resveratrol	10	400	19.8
ZmUGT72G4	Resveratrol	10	200	36.8
ZmUGT72G4	Resveratrol	10	100	67
ZmUGT72G4	Resveratrol	10	50	85.3
ZmUGT72G4	Resveratrol	20	400	39.5
ZmUGT72G4	Resveratrol	20	200	65.4
ZmUGT72G4	Resveratrol	20	100	93.6
ZmUGT72G4	Resveratrol	20	50	99.4
ZmUGT72G4	Resveratrol	40	400	43.7
ZmUGT72G4	Resveratrol	40	200	90.5
ZmUGT72G4	Resveratrol	40	100	99.7
ZmUGT72G4	Resveratrol	40	50	100
ZmUGT72G4	Resveratrol	80	400	75.5
ZmUGT72G4	Resveratrol	80	200	98.1
ZmUGT72G4	Resveratrol	80	100	99.9
ZmUGT72G4	Resveratrol	80	50	100
ZmUGT708A6	Resveratrol	10	400	0.3
ZmUGT708A6	Resveratrol	10	200	0.5
ZmUGT708A6	Resveratrol	10	100	1.1
ZmUGT708A6	Resveratrol	10	50	1.8
ZmUGT708A6	Resveratrol	20	400	0.7
ZmUGT708A6	Resveratrol	20	200	2.6
ZmUGT708A6	Resveratrol	20	100	3.1
ZmUGT708A6	Resveratrol	20	50	8.6
ZmUGT708A6	Resveratrol	40	400	3.3
ZmUGT708A6	Resveratrol	40	200	5.9
ZmUGT708A6	Resveratrol	40	100	10.2
ZmUGT708A6	Resveratrol	40	50	18.3
ZmUGT708A6	Resveratrol	80	400	8.9
ZmUGT708A6	Resveratrol	80	200	11.2
ZmUGT708A6	Resveratrol	80	100	20.5
ZmUGT708A6	Resveratrol	80	50	38.6
ZmUGT88C10	Resveratrol	10	400	0.2
ZmUGT88C10	Resveratrol	10	200	1.1
ZmUGT88C10	Resveratrol	10	100	4.1
ZmUGT88C10	Resveratrol	10	50	8.1
ZmUGT88C10	Resveratrol	20	400	1.5
ZmUGT88C10	Resveratrol	20	200	4.5
ZmUGT88C10	Resveratrol	20	100	9
ZmUGT88C10	Resveratrol	20	50	17.5
ZmUGT88C10	Resveratrol	40	400	5.4

ZmUGT88C10	Resveratrol	40	200	10.9
ZmUGT88C10	Resveratrol	40	100	18
ZmUGT88C10	Resveratrol	40	50	34
ZmUGT88C10	Resveratrol	80	400	12.7
ZmUGT88C10	Resveratrol	80	200	20.4
ZmUGT88C10	Resveratrol	80	100	33.9
ZmUGT88C10	Resveratrol	80	50	59.4
ZmUGT706F8	Resveratrol	10	400	0
ZmUGT706F8	Resveratrol	10	200	0.2
ZmUGT706F8	Resveratrol	10	100	0.6
ZmUGT706F8	Resveratrol	10	50	3.6
ZmUGT706F8	Resveratrol	20	400	0.5
ZmUGT706F8	Resveratrol	20	200	0.7
ZmUGT706F8	Resveratrol	20	100	3.4
ZmUGT706F8	Resveratrol	20	50	7.3
ZmUGT706F8	Resveratrol	40	400	1.1
ZmUGT706F8	Resveratrol	40	200	3.4
ZmUGT706F8	Resveratrol	40	100	8.6
ZmUGT706F8	Resveratrol	40	50	18.2
ZmUGT706F8	Resveratrol	80	400	3.4
ZmUGT706F8	Resveratrol	80	200	6.4
ZmUGT706F8	Resveratrol	80	100	19
ZmUGT706F8	Resveratrol	80	50	36.8
Zm71B1	Resveratrol	10	400	9.2
Zm71B1	Resveratrol	10	200	15.9
Zm71B1	Resveratrol	10	100	34
Zm71B1	Resveratrol	10	50	67.3
Zm71B1	Resveratrol	20	400	23
Zm71B1	Resveratrol	20	200	38.4
Zm71B1	Resveratrol	20	100	74.7
Zm71B1	Resveratrol	20	50	99.1
Zm71B1	Resveratrol	40	400	41
Zm71B1	Resveratrol	40	200	71.3
Zm71B1	Resveratrol	40	100	97.5
Zm71B1	Resveratrol	40	50	99.7
Zm71B1	Resveratrol	80	400	68.3
Zm71B1	Resveratrol	80	200	95.7
Zm71B1	Resveratrol	80	100	98.4
Zm71B1	Resveratrol	80	50	99.7
SlUGT72B68	Resveratrol	10	400	95.5
SlUGT72B68	Resveratrol	10	200	98.9
SlUGT72B68	Resveratrol	10	100	99.2
SlUGT72B68	Resveratrol	10	50	100

SlUGT72B68	Resveratrol	20	400	94.5
SlUGT72B68	Resveratrol	20	200	98.6
SlUGT72B68	Resveratrol	20	100	99.4
SlUGT72B68	Resveratrol	20	50	100
SlUGT72B68	Resveratrol	40	400	92.9
SlUGT72B68	Resveratrol	40	200	97.4
SlUGT72B68	Resveratrol	40	100	NA
SlUGT72B68	Resveratrol	40	50	100
SlUGT72B68	Resveratrol	80	400	91.8
SlUGT72B68	Resveratrol	80	200	96.5
SlUGT72B68	Resveratrol	80	100	98.9
SlUGT72B68	Resveratrol	80	50	100
RhGT1	Resveratrol	10	400	58.8
RhGT1	Resveratrol	10	200	89.9
RhGT1	Resveratrol	10	100	100
RhGT1	Resveratrol	10	50	100
RhGT1	Resveratrol	20	400	80.3
RhGT1	Resveratrol	20	200	98.7
RhGT1	Resveratrol	20	100	100
RhGT1	Resveratrol	20	50	100
RhGT1	Resveratrol	40	400	95.4
RhGT1	Resveratrol	40	200	98.6
RhGT1	Resveratrol	40	100	100
RhGT1	Resveratrol	40	50	100
RhGT1	Resveratrol	80	400	95.9
RhGT1	Resveratrol	80	200	98.2
RhGT1	Resveratrol	80	100	100
RhGT1	Resveratrol	80	50	100
Os88C1	Resveratrol	10	400	10
Os88C1	Resveratrol	10	200	16.6
Os88C1	Resveratrol	10	100	25.9
Os88C1	Resveratrol	10	50	37.9
Os88C1	Resveratrol	20	400	15.4
Os88C1	Resveratrol	20	200	29.1
Os88C1	Resveratrol	20	100	48.2
Os88C1	Resveratrol	20	50	68.1
Os88C1	Resveratrol	40	400	24.9
Os88C1	Resveratrol	40	200	45.3
Os88C1	Resveratrol	40	100	79.4
Os88C1	Resveratrol	40	50	97
Os88C1	Resveratrol	80	400	26.8
Os88C1	Resveratrol	80	200	73.2
Os88C1	Resveratrol	80	100	98.5
Os88C1	Resveratrol	80	50	100

Lc72B10	Resveratrol	10	400	22
Lc72B10	Resveratrol	10	200	53.7
Lc72B10	Resveratrol	10	100	85.5
Lc72B10	Resveratrol	10	50	99
Lc72B10	Resveratrol	20	400	53
Lc72B10	Resveratrol	20	200	85
Lc72B10	Resveratrol	20	100	99.3
Lc72B10	Resveratrol	20	50	100
Lc72B10	Resveratrol	40	400	68
Lc72B10	Resveratrol	40	200	97.7
Lc72B10	Resveratrol	40	100	100
Lc72B10	Resveratrol	40	50	100
Lc72B10	Resveratrol	80	400	85.2
Lc72B10	Resveratrol	80	200	98.4
Lc72B10	Resveratrol	80	100	100
Lc72B10	Resveratrol	80	50	100
Gm88E3	Resveratrol	10	400	89.9
Gm88E3	Resveratrol	10	200	98
Gm88E3	Resveratrol	10	100	98.5
Gm88E3	Resveratrol	10	50	100
Gm88E3	Resveratrol	20	400	93.5
Gm88E3	Resveratrol	20	200	97.8
Gm88E3	Resveratrol	20	100	98.8
Gm88E3	Resveratrol	20	50	100
Gm88E3	Resveratrol	40	400	91.9
Gm88E3	Resveratrol	40	200	97.5
Gm88E3	Resveratrol	40	100	98.9
Gm88E3	Resveratrol	40	50	100
Gm88E3	Resveratrol	80	400	91.8
Gm88E3	Resveratrol	80	200	97.5
Gm88E3	Resveratrol	80	100	98.8
Gm88E3	Resveratrol	80	50	100
Fi88A10	Resveratrol	10	400	29.4
Fi88A10	Resveratrol	10	200	54.4
Fi88A10	Resveratrol	10	100	95.3
Fi88A10	Resveratrol	10	50	100
Fi88A10	Resveratrol	20	400	55.4
Fi88A10	Resveratrol	20	200	93.4
Fi88A10	Resveratrol	20	100	99.7
Fi88A10	Resveratrol	20	50	100
Fi88A10	Resveratrol	40	400	76.8
Fi88A10	Resveratrol	40	200	98.8
Fi88A10	Resveratrol	40	100	95.8

Fi88A10	Resveratrol	40	50	100
Fi88A10	Resveratrol	80	400	NA
Fi88A10	Resveratrol	80	200	98.5
Fi88A10	Resveratrol	80	100	99.5
Fi88A10	Resveratrol	80	50	100
Fe88J1	Resveratrol	10	400	4.5
Fe88J1	Resveratrol	10	200	7.5
Fe88J1	Resveratrol	10	100	12
Fe88J1	Resveratrol	10	50	19.7
Fe88J1	Resveratrol	20	400	5.4
Fe88J1	Resveratrol	20	200	12.1
Fe88J1	Resveratrol	20	100	22.6
Fe88J1	Resveratrol	20	50	39.3
Fe88J1	Resveratrol	40	400	19.5
Fe88J1	Resveratrol	40	200	23.5
Fe88J1	Resveratrol	40	100	42.5
Fe88J1	Resveratrol	40	50	72.4
Fe88J1	Resveratrol	80	400	18.8
Fe88J1	Resveratrol	80	200	43.3
Fe88J1	Resveratrol	80	100	78.7
Fe88J1	Resveratrol	80	50	99.8
At71D1	Resveratrol	10	400	14.5
At71D1	Resveratrol	10	200	27.2
At71D1	Resveratrol	10	100	49.5
At71D1	Resveratrol	10	50	76
At71D1	Resveratrol	20	400	22.2
At71D1	Resveratrol	20	200	40.5
At71D1	Resveratrol	20	100	71.2
At71D1	Resveratrol	20	50	94
At71D1	Resveratrol	40	400	33
At71D1	Resveratrol	40	200	58.1
At71D1	Resveratrol	40	100	89.9
At71D1	Resveratrol	40	50	98.9
At71D1	Resveratrol	80	400	49.8
At71D1	Resveratrol	80	200	76.5
At71D1	Resveratrol	80	100	98.3
At71D1	Resveratrol	80	50	100
At71C1	Resveratrol	10	400	17.2
At71C1	Resveratrol	10	200	32.1
At71C1	Resveratrol	10	100	56
At71C1	Resveratrol	10	50	78.9
At71C1	Resveratrol	20	400	27.5
At71C1	Resveratrol	20	200	52.8
At71C1	Resveratrol	20	100	85.3

At71C1	Resveratrol	20	50	98
At71C1	Resveratrol	40	400	31.2
At71C1	Resveratrol	40	200	74.3
At71C1	Resveratrol	40	100	97.7
At71C1	Resveratrol	40	50	100
At71C1	Resveratrol	80	400	60.3
At71C1	Resveratrol	80	200	92.7
At71C1	Resveratrol	80	100	98.9
At71C1	Resveratrol	80	50	100
PtUGT1	Resveratrol	10	400	1.8
PtUGT1	Resveratrol	10	200	6.7
PtUGT1	Resveratrol	10	100	10.8
PtUGT1	Resveratrol	10	50	18
PtUGT1	Resveratrol	20	400	6.8
PtUGT1	Resveratrol	20	200	15.7
PtUGT1	Resveratrol	20	100	27.2
PtUGT1	Resveratrol	20	50	46.9
PtUGT1	Resveratrol	40	400	16.3
PtUGT1	Resveratrol	40	200	33.5
PtUGT1	Resveratrol	40	100	63.3
PtUGT1	Resveratrol	40	50	92
PtUGT1	Resveratrol	80	400	34.8
PtUGT1	Resveratrol	80	200	60.3
PtUGT1	Resveratrol	80	100	92.8
PtUGT1	Resveratrol	80	50	100
AtUGT72E2	Resveratrol	10	400	0
AtUGT72E2	Resveratrol	10	200	0
AtUGT72E2	Resveratrol	10	100	0
AtUGT72E2	Resveratrol	10	50	0
AtUGT72E2	Resveratrol	20	400	0
AtUGT72E2	Resveratrol	20	200	0
AtUGT72E2	Resveratrol	20	100	0.6
AtUGT72E2	Resveratrol	20	50	2.4
AtUGT72E2	Resveratrol	40	400	0
AtUGT72E2	Resveratrol	40	200	1
AtUGT72E2	Resveratrol	40	100	3.4
AtUGT72E2	Resveratrol	40	50	8.5
AtUGT72E2	Resveratrol	80	400	1.2
AtUGT72E2	Resveratrol	80	200	4.4
AtUGT72E2	Resveratrol	80	100	8.1
AtUGT72E2	Resveratrol	80	50	15.7
MtUGT78G1	Resveratrol	10	400	23.8
MtUGT78G1	Resveratrol	10	200	42.9

MtUGT78G1	Resveratrol	10	100	65.9
MtUGT78G1	Resveratrol	10	50	83.5
MtUGT78G1	Resveratrol	20	400	24.1
MtUGT78G1	Resveratrol	20	200	58.7
MtUGT78G1	Resveratrol	20	100	81.6
MtUGT78G1	Resveratrol	20	50	92.7
MtUGT78G1	Resveratrol	40	400	38.1
MtUGT78G1	Resveratrol	40	200	73.9
MtUGT78G1	Resveratrol	40	100	90.6
MtUGT78G1	Resveratrol	40	50	96.2
MtUGT78G1	Resveratrol	80	400	59.4
MtUGT78G1	Resveratrol	80	200	86
MtUGT78G1	Resveratrol	80	100	94.9
MtUGT78G1	Resveratrol	80	50	97.6
ZmUGT72G3	Scopoletin	10	400	31.8
ZmUGT72G3	Scopoletin	10	200	58.5
ZmUGT72G3	Scopoletin	10	100	76.9
ZmUGT72G3	Scopoletin	10	50	88
ZmUGT72G3	Scopoletin	20	400	45.5
ZmUGT72G3	Scopoletin	20	200	68.2
ZmUGT72G3	Scopoletin	20	100	83.3
ZmUGT72G3	Scopoletin	20	50	90.6
ZmUGT72G3	Scopoletin	40	400	51.7
ZmUGT72G3	Scopoletin	40	200	71.4
ZmUGT72G3	Scopoletin	40	100	84
ZmUGT72G3	Scopoletin	40	50	89.8
ZmUGT72G3	Scopoletin	80	400	52.9
ZmUGT72G3	Scopoletin	80	200	70.9
ZmUGT72G3	Scopoletin	80	100	82.7
ZmUGT72G3	Scopoletin	80	50	87.9
ZmUGT72G4	Scopoletin	10	400	43.5
ZmUGT72G4	Scopoletin	10	200	65.2
ZmUGT72G4	Scopoletin	10	100	80
ZmUGT72G4	Scopoletin	10	50	88.5
ZmUGT72G4	Scopoletin	20	400	53
ZmUGT72G4	Scopoletin	20	200	72.6
ZmUGT72G4	Scopoletin	20	100	84.8
ZmUGT72G4	Scopoletin	20	50	91.8
ZmUGT72G4	Scopoletin	40	400	55.2
ZmUGT72G4	Scopoletin	40	200	73.1
ZmUGT72G4	Scopoletin	40	100	85.2
ZmUGT72G4	Scopoletin	40	50	90.9
ZmUGT72G4	Scopoletin	80	400	56.6
ZmUGT72G4	Scopoletin	80	200	73.3

ZmUGT72G4	Scopoletin	80	100	83.9
ZmUGT72G4	Scopoletin	80	50	89.6
ZmUGT708A6	Scopoletin	10	400	0
ZmUGT708A6	Scopoletin	10	200	0
ZmUGT708A6	Scopoletin	10	100	0
ZmUGT708A6	Scopoletin	10	50	0.8
ZmUGT708A6	Scopoletin	20	400	0
ZmUGT708A6	Scopoletin	20	200	1.1
ZmUGT708A6	Scopoletin	20	100	2.1
ZmUGT708A6	Scopoletin	20	50	2.8
ZmUGT708A6	Scopoletin	40	400	2.3
ZmUGT708A6	Scopoletin	40	200	2.7
ZmUGT708A6	Scopoletin	40	100	6.9
ZmUGT708A6	Scopoletin	40	50	10.6
ZmUGT708A6	Scopoletin	80	400	5.4
ZmUGT708A6	Scopoletin	80	200	7
ZmUGT708A6	Scopoletin	80	100	16.1
ZmUGT708A6	Scopoletin	80	50	29.3
ZmUGT88C10	Scopoletin	10	400	0
ZmUGT88C10	Scopoletin	10	200	0
ZmUGT88C10	Scopoletin	10	100	0
ZmUGT88C10	Scopoletin	10	50	0
ZmUGT88C10	Scopoletin	20	400	0
ZmUGT88C10	Scopoletin	20	200	1.8
ZmUGT88C10	Scopoletin	20	100	1.6
ZmUGT88C10	Scopoletin	20	50	1.5
ZmUGT88C10	Scopoletin	40	400	2.5
ZmUGT88C10	Scopoletin	40	200	2.8
ZmUGT88C10	Scopoletin	40	100	8
ZmUGT88C10	Scopoletin	40	50	12.2
ZmUGT88C10	Scopoletin	80	400	5.2
ZmUGT88C10	Scopoletin	80	200	7
ZmUGT88C10	Scopoletin	80	100	14
ZmUGT88C10	Scopoletin	80	50	18.1
ZmUGT706F8	Scopoletin	10	400	4.4
ZmUGT706F8	Scopoletin	10	200	18
ZmUGT706F8	Scopoletin	10	100	26.5
ZmUGT706F8	Scopoletin	10	50	38.2
ZmUGT706F8	Scopoletin	20	400	13.4
ZmUGT706F8	Scopoletin	20	200	35.1
ZmUGT706F8	Scopoletin	20	100	47.9
ZmUGT706F8	Scopoletin	20	50	66.4
ZmUGT706F8	Scopoletin	40	400	25.6

ZmUGT706F8	Scopoletin	40	200	52.9
ZmUGT706F8	Scopoletin	40	100	68.6
ZmUGT706F8	Scopoletin	40	50	85.4
ZmUGT706F8	Scopoletin	80	400	37.6
ZmUGT706F8	Scopoletin	80	200	66.9
ZmUGT706F8	Scopoletin	80	100	80.9
ZmUGT706F8	Scopoletin	80	50	90.4
Zm71B1	Scopoletin	10	400	1.2
Zm71B1	Scopoletin	10	200	4.9
Zm71B1	Scopoletin	10	100	14.8
Zm71B1	Scopoletin	10	50	23.7
Zm71B1	Scopoletin	20	400	7.2
Zm71B1	Scopoletin	20	200	18
Zm71B1	Scopoletin	20	100	26.2
Zm71B1	Scopoletin	20	50	38.5
Zm71B1	Scopoletin	40	400	19.2
Zm71B1	Scopoletin	40	200	28.7
Zm71B1	Scopoletin	40	100	39.4
Zm71B1	Scopoletin	40	50	56
Zm71B1	Scopoletin	80	400	28.7
Zm71B1	Scopoletin	80	200	41.8
Zm71B1	Scopoletin	80	100	56.3
Zm71B1	Scopoletin	80	50	74.4
SlUGT72B68	Scopoletin	10	400	24.4
SlUGT72B68	Scopoletin	10	200	45
SlUGT72B68	Scopoletin	10	100	69.4
SlUGT72B68	Scopoletin	10	50	88.1
SlUGT72B68	Scopoletin	20	400	25.2
SlUGT72B68	Scopoletin	20	200	50.6
SlUGT72B68	Scopoletin	20	100	74.9
SlUGT72B68	Scopoletin	20	50	86
SlUGT72B68	Scopoletin	40	400	27.2
SlUGT72B68	Scopoletin	40	200	52
SlUGT72B68	Scopoletin	40	100	74.7
SlUGT72B68	Scopoletin	40	50	81.8
SlUGT72B68	Scopoletin	80	400	32.2
SlUGT72B68	Scopoletin	80	200	57.4
SlUGT72B68	Scopoletin	80	100	70.6
SlUGT72B68	Scopoletin	80	50	74
RhGT1	Scopoletin	10	400	15.7
RhGT1	Scopoletin	10	200	30.4
RhGT1	Scopoletin	10	100	47.4
RhGT1	Scopoletin	10	50	69.2
RhGT1	Scopoletin	20	400	NA

RhGT1	Scopoletin	20	200	NA
RhGT1	Scopoletin	20	100	NA
RhGT1	Scopoletin	20	50	NA
RhGT1	Scopoletin	40	400	32
RhGT1	Scopoletin	40	200	57
RhGT1	Scopoletin	40	100	77.4
RhGT1	Scopoletin	40	50	88.8
RhGT1	Scopoletin	80	400	37.2
RhGT1	Scopoletin	80	200	63.6
RhGT1	Scopoletin	80	100	80.9
RhGT1	Scopoletin	80	50	88.9
Os88C1	Scopoletin	10	400	0.9
Os88C1	Scopoletin	10	200	6.5
Os88C1	Scopoletin	10	100	13.2
Os88C1	Scopoletin	10	50	16.2
Os88C1	Scopoletin	20	400	7.5
Os88C1	Scopoletin	20	200	15.4
Os88C1	Scopoletin	20	100	21
Os88C1	Scopoletin	20	50	25.6
Os88C1	Scopoletin	40	400	12.1
Os88C1	Scopoletin	40	200	NA
Os88C1	Scopoletin	40	100	28.8
Os88C1	Scopoletin	40	50	35.9
Os88C1	Scopoletin	80	400	17.5
Os88C1	Scopoletin	80	200	28.5
Os88C1	Scopoletin	80	100	39.7
Os88C1	Scopoletin	80	50	47.5
Lc72B10	Scopoletin	10	400	2.8
Lc72B10	Scopoletin	10	200	8.9
Lc72B10	Scopoletin	10	100	20
Lc72B10	Scopoletin	10	50	35
Lc72B10	Scopoletin	20	400	4.8
Lc72B10	Scopoletin	20	200	18.6
Lc72B10	Scopoletin	20	100	32.3
Lc72B10	Scopoletin	20	50	50.1
Lc72B10	Scopoletin	40	400	15
Lc72B10	Scopoletin	40	200	28.6
Lc72B10	Scopoletin	40	100	49.7
Lc72B10	Scopoletin	40	50	65.4
Lc72B10	Scopoletin	80	400	26.6
Lc72B10	Scopoletin	80	200	46.9
Lc72B10	Scopoletin	80	100	64.7
Lc72B10	Scopoletin	80	50	75.7

Gm88E3	Scopoletin	10	400	0.8
Gm88E3	Scopoletin	10	200	7.1
Gm88E3	Scopoletin	10	100	14.2
Gm88E3	Scopoletin	10	50	23.2
Gm88E3	Scopoletin	20	400	NA
Gm88E3	Scopoletin	20	200	NA
Gm88E3	Scopoletin	20	100	NA
Gm88E3	Scopoletin	20	50	NA
Gm88E3	Scopoletin	40	400	11.2
Gm88E3	Scopoletin	40	200	22.8
Gm88E3	Scopoletin	40	100	36
Gm88E3	Scopoletin	40	50	55.2
Gm88E3	Scopoletin	80	400	16.9
Gm88E3	Scopoletin	80	200	32.3
Gm88E3	Scopoletin	80	100	50.1
Gm88E3	Scopoletin	80	50	71.1
Fi88A10	Scopoletin	10	400	37.9
Fi88A10	Scopoletin	10	200	62.9
Fi88A10	Scopoletin	10	100	80
Fi88A10	Scopoletin	10	50	90.1
Fi88A10	Scopoletin	20	400	43.9
Fi88A10	Scopoletin	20	200	71.5
Fi88A10	Scopoletin	20	100	82.3
Fi88A10	Scopoletin	20	50	90
Fi88A10	Scopoletin	40	400	41.8
Fi88A10	Scopoletin	40	200	68.4
Fi88A10	Scopoletin	40	100	80.8
Fi88A10	Scopoletin	40	50	88.8
Fi88A10	Scopoletin	80	400	41.6
Fi88A10	Scopoletin	80	200	68.1
Fi88A10	Scopoletin	80	100	78.9
Fi88A10	Scopoletin	80	50	85.8
Fe88J1	Scopoletin	10	400	0
Fe88J1	Scopoletin	10	200	0
Fe88J1	Scopoletin	10	100	0
Fe88J1	Scopoletin	10	50	0
Fe88J1	Scopoletin	20	400	0
Fe88J1	Scopoletin	20	200	0
Fe88J1	Scopoletin	20	100	0.9
Fe88J1	Scopoletin	20	50	2.1
Fe88J1	Scopoletin	40	400	0
Fe88J1	Scopoletin	40	200	1
Fe88J1	Scopoletin	40	100	1.2
Fe88J1	Scopoletin	40	50	2.9

Fe88J1	Scopoletin	80	400	1.3
Fe88J1	Scopoletin	80	200	1.6
Fe88J1	Scopoletin	80	100	3.2
Fe88J1	Scopoletin	80	50	3.9
At71D1	Scopoletin	10	400	9.4
At71D1	Scopoletin	10	200	15.2
At71D1	Scopoletin	10	100	21.5
At71D1	Scopoletin	10	50	26.9
At71D1	Scopoletin	20	400	14.8
At71D1	Scopoletin	20	200	23.6
At71D1	Scopoletin	20	100	32.9
At71D1	Scopoletin	20	50	40.4
At71D1	Scopoletin	40	400	20.4
At71D1	Scopoletin	40	200	32.1
At71D1	Scopoletin	40	100	45.1
At71D1	Scopoletin	40	50	54.6
At71D1	Scopoletin	80	400	27.8
At71D1	Scopoletin	80	200	42.8
At71D1	Scopoletin	80	100	57.6
At71D1	Scopoletin	80	50	69.1
At71C1	Scopoletin	10	400	44.9
At71C1	Scopoletin	10	200	63.8
At71C1	Scopoletin	10	100	78.9
At71C1	Scopoletin	10	50	87.2
At71C1	Scopoletin	20	400	46.5
At71C1	Scopoletin	20	200	63.6
At71C1	Scopoletin	20	100	78
At71C1	Scopoletin	20	50	86.4
At71C1	Scopoletin	40	400	48.7
At71C1	Scopoletin	40	200	65.2
At71C1	Scopoletin	40	100	79.5
At71C1	Scopoletin	40	50	87.1
At71C1	Scopoletin	80	400	49.3
At71C1	Scopoletin	80	200	66.9
At71C1	Scopoletin	80	100	80.9
At71C1	Scopoletin	80	50	87.9
PtUGT1	Scopoletin	10	400	4.8
PtUGT1	Scopoletin	10	200	14
PtUGT1	Scopoletin	10	100	28.2
PtUGT1	Scopoletin	10	50	55.9
PtUGT1	Scopoletin	20	400	15.9
PtUGT1	Scopoletin	20	200	34.1
PtUGT1	Scopoletin	20	100	61.6

PtUGT1	Scopoletin	20	50	88.1
PtUGT1	Scopoletin	40	400	33.2
PtUGT1	Scopoletin	40	200	58.3
PtUGT1	Scopoletin	40	100	78
PtUGT1	Scopoletin	40	50	87.6
PtUGT1	Scopoletin	80	400	43.5
PtUGT1	Scopoletin	80	200	65.1
PtUGT1	Scopoletin	80	100	76.6
PtUGT1	Scopoletin	80	50	84.8
AtUGT72E2	Scopoletin	10	400	1
AtUGT72E2	Scopoletin	10	200	2.2
AtUGT72E2	Scopoletin	10	100	3
AtUGT72E2	Scopoletin	10	50	5.2
AtUGT72E2	Scopoletin	20	400	4.6
AtUGT72E2	Scopoletin	20	200	7.4
AtUGT72E2	Scopoletin	20	100	10.5
AtUGT72E2	Scopoletin	20	50	15.1
AtUGT72E2	Scopoletin	40	400	11.5
AtUGT72E2	Scopoletin	40	200	22.4
AtUGT72E2	Scopoletin	40	100	31.7
AtUGT72E2	Scopoletin	40	50	47.8
AtUGT72E2	Scopoletin	80	400	26.3
AtUGT72E2	Scopoletin	80	200	51.3
AtUGT72E2	Scopoletin	80	100	69.4
AtUGT72E2	Scopoletin	80	50	87.6
MtUGT78G1	Scopoletin	10	400	3.7
MtUGT78G1	Scopoletin	10	200	5.8
MtUGT78G1	Scopoletin	10	100	8.3
MtUGT78G1	Scopoletin	10	50	13
MtUGT78G1	Scopoletin	20	400	5.9
MtUGT78G1	Scopoletin	20	200	9.2
MtUGT78G1	Scopoletin	20	100	13
MtUGT78G1	Scopoletin	20	50	19.9
MtUGT78G1	Scopoletin	40	400	8.9
MtUGT78G1	Scopoletin	40	200	13.7
MtUGT78G1	Scopoletin	40	100	19.6
MtUGT78G1	Scopoletin	40	50	29.3
MtUGT78G1	Scopoletin	80	400	12.2
MtUGT78G1	Scopoletin	80	200	20
MtUGT78G1	Scopoletin	80	100	29.4
MtUGT78G1	Scopoletin	80	50	43.7
ZmUGT72G3	Scopoletin	0	400	0
ZmUGT72G3	Scopoletin	0	200	0
ZmUGT72G3	Scopoletin	0	100	0

ZmUGT72G3	Scopoletin	0	50	0
ZmUGT72G3	Apigenin	0	400	0
ZmUGT72G3	Apigenin	0	200	0
ZmUGT72G3	Apigenin	0	100	0
ZmUGT72G3	Apigenin	0	50	0
ZmUGT72G3	Resveratrol	0	400	0
ZmUGT72G3	Resveratrol	0	200	0
ZmUGT72G3	Resveratrol	0	100	0
ZmUGT72G3	Resveratrol	0	50	0
ZmUGT72G4	Scopoletin	0	400	0
ZmUGT72G4	Scopoletin	0	200	0
ZmUGT72G4	Scopoletin	0	100	0
ZmUGT72G4	Scopoletin	0	50	0
ZmUGT72G4	Apigenin	0	400	0
ZmUGT72G4	Apigenin	0	200	0
ZmUGT72G4	Apigenin	0	100	0
ZmUGT72G4	Apigenin	0	50	0
ZmUGT72G4	Resveratrol	0	400	0
ZmUGT72G4	Resveratrol	0	200	0
ZmUGT72G4	Resveratrol	0	100	0
ZmUGT72G4	Resveratrol	0	50	0
ZmUGT708A6	Scopoletin	0	400	0
ZmUGT708A6	Scopoletin	0	200	0
ZmUGT708A6	Scopoletin	0	100	0
ZmUGT708A6	Scopoletin	0	50	0
ZmUGT708A6	Apigenin	0	400	0
ZmUGT708A6	Apigenin	0	200	0
ZmUGT708A6	Apigenin	0	100	0
ZmUGT708A6	Apigenin	0	50	0
ZmUGT708A6	Resveratrol	0	400	0
ZmUGT708A6	Resveratrol	0	200	0
ZmUGT708A6	Resveratrol	0	100	0
ZmUGT708A6	Resveratrol	0	50	0
ZmUGT88C10	Scopoletin	0	400	0
ZmUGT88C10	Scopoletin	0	200	0
ZmUGT88C10	Scopoletin	0	100	0
ZmUGT88C10	Scopoletin	0	50	0
ZmUGT88C10	Apigenin	0	400	0
ZmUGT88C10	Apigenin	0	200	0
ZmUGT88C10	Apigenin	0	100	0
ZmUGT88C10	Apigenin	0	50	0
ZmUGT88C10	Resveratrol	0	400	0
ZmUGT88C10	Resveratrol	0	200	0

ZmUGT88C10	Resveratrol	0	100	0
ZmUGT88C10	Resveratrol	0	50	0
ZmUGT706F8	Scopoletin	0	400	0
ZmUGT706F8	Scopoletin	0	200	0
ZmUGT706F8	Scopoletin	0	100	0
ZmUGT706F8	Scopoletin	0	50	0
ZmUGT706F8	Apigenin	0	400	0
ZmUGT706F8	Apigenin	0	200	0
ZmUGT706F8	Apigenin	0	100	0
ZmUGT706F8	Apigenin	0	50	0
ZmUGT706F8	Resveratrol	0	400	0
ZmUGT706F8	Resveratrol	0	200	0
ZmUGT706F8	Resveratrol	0	100	0
ZmUGT706F8	Resveratrol	0	50	0
ZmUGT706F8	Scopoletin	0	400	0
Zm71B1	Scopoletin	0	200	0
Zm71B1	Scopoletin	0	100	0
Zm71B1	Scopoletin	0	50	0
Zm71B1	Apigenin	0	400	0
Zm71B1	Apigenin	0	200	0
Zm71B1	Apigenin	0	100	0
Zm71B1	Apigenin	0	50	0
Zm71B1	Resveratrol	0	400	0
Zm71B1	Resveratrol	0	200	0
Zm71B1	Resveratrol	0	100	0
Zm71B1	Resveratrol	0	50	0
PtUGT1	Scopoletin	0	400	0
PtUGT1	Scopoletin	0	200	0
PtUGT1	Scopoletin	0	100	0
PtUGT1	Scopoletin	0	50	0
PtUGT1	Apigenin	0	400	0
PtUGT1	Apigenin	0	200	0
PtUGT1	Apigenin	0	100	0
PtUGT1	Apigenin	0	50	0
PtUGT1	Resveratrol	0	400	0
PtUGT1	Resveratrol	0	200	0
PtUGT1	Resveratrol	0	100	0
PtUGT1	Resveratrol	0	50	0
AtUGT72E2	Scopoletin	0	400	0
AtUGT72E2	Scopoletin	0	200	0
AtUGT72E2	Scopoletin	0	100	0
AtUGT72E2	Scopoletin	0	50	0
AtUGT72E2	Apigenin	0	400	0
AtUGT72E2	Apigenin	0	200	0

AtUGT72E2	Apigenin	0	100	0
AtUGT72E2	Apigenin	0	50	0
AtUGT72E2	Resveratrol	0	400	0
AtUGT72E2	Resveratrol	0	200	0
AtUGT72E2	Resveratrol	0	100	0
AtUGT72E2	Resveratrol	0	50	0
MtUGT78G1	Scopoletin	0	400	0
MtUGT78G1	Scopoletin	0	200	0
MtUGT78G1	Scopoletin	0	100	0
MtUGT78G1	Scopoletin	0	50	0
MtUGT78G1	Apigenin	0	400	0
MtUGT78G1	Apigenin	0	200	0
MtUGT78G1	Apigenin	0	100	0
MtUGT78G1	Apigenin	0	50	0
MtUGT78G1	Resveratrol	0	400	0
MtUGT78G1	Resveratrol	0	200	0
MtUGT78G1	Resveratrol	0	100	0
MtUGT78G1	Resveratrol	0	50	0
SlUGT72B68	Scopoletin	0	400	0
SlUGT72B68	Scopoletin	0	200	0
SlUGT72B68	Scopoletin	0	100	0
SlUGT72B68	Scopoletin	0	50	0
SlUGT72B68	Apigenin	0	400	0
SlUGT72B68	Apigenin	0	200	0
SlUGT72B68	Apigenin	0	100	0
SlUGT72B68	Apigenin	0	50	0
SlUGT72B68	Resveratrol	0	400	0
SlUGT72B68	Resveratrol	0	200	0
SlUGT72B68	Resveratrol	0	100	0
SlUGT72B68	Resveratrol	0	50	0
RhGT1	Scopoletin	0	400	0
RhGT1	Scopoletin	0	200	0
RhGT1	Scopoletin	0	100	0
RhGT1	Scopoletin	0	50	0
RhGT1	Apigenin	0	400	0
RhGT1	Apigenin	0	200	0
RhGT1	Apigenin	0	100	0
RhGT1	Apigenin	0	50	0
RhGT1	Resveratrol	0	400	0
RhGT1	Resveratrol	0	200	0
RhGT1	Resveratrol	0	100	0
RhGT1	Resveratrol	0	50	0
Os88C1	Scopoletin	0	400	0

Os88C1	Scopoletin	0	200	0
Os88C1	Scopoletin	0	100	0
Os88C1	Scopoletin	0	50	0
Os88C1	Apigenin	0	400	0
Os88C1	Apigenin	0	200	0
Os88C1	Apigenin	0	100	0
Os88C1	Apigenin	0	50	0
Os88C1	Resveratrol	0	400	0
Os88C1	Resveratrol	0	200	0
Os88C1	Resveratrol	0	100	0
Os88C1	Resveratrol	0	50	0
Lc72B10	Scopoletin	0	400	0
Lc72B10	Scopoletin	0	200	0
Lc72B10	Scopoletin	0	100	0
Lc72B10	Scopoletin	0	50	0
Lc72B10	Apigenin	0	400	0
Lc72B10	Apigenin	0	200	0
Lc72B10	Apigenin	0	100	0
Lc72B10	Apigenin	0	50	0
Lc72B10	Resveratrol	0	400	0
Lc72B10	Resveratrol	0	200	0
Lc72B10	Resveratrol	0	100	0
Lc72B10	Resveratrol	0	50	0
Gm88E3	Scopoletin	0	400	0
Gm88E3	Scopoletin	0	200	0
Gm88E3	Scopoletin	0	100	0
Gm88E3	Scopoletin	0	50	0
Gm88E3	Apigenin	0	400	0
Gm88E3	Apigenin	0	200	0
Gm88E3	Apigenin	0	100	0
Gm88E3	Apigenin	0	50	0
Gm88E3	Resveratrol	0	400	0
Gm88E3	Resveratrol	0	200	0
Gm88E3	Resveratrol	0	100	0
Gm88E3	Resveratrol	0	50	0
Fi88A10	Scopoletin	0	400	0
Fi88A10	Scopoletin	0	200	0
Fi88A10	Scopoletin	0	100	0
Fi88A10	Scopoletin	0	50	0
Fi88A10	Apigenin	0	400	0
Fi88A10	Apigenin	0	200	0
Fi88A10	Apigenin	0	100	0
Fi88A10	Apigenin	0	50	0
Fi88A10	Resveratrol	0	400	0

Fi88A10	Resveratrol	0	200	0
Fi88A10	Resveratrol	0	100	0
Fi88A10	Resveratrol	0	50	0
Fe88J1	Scopoletin	0	400	0
Fe88J1	Scopoletin	0	200	0
Fe88J1	Scopoletin	0	100	0
Fe88J1	Scopoletin	0	50	0
Fe88J1	Apigenin	0	400	0
Fe88J1	Apigenin	0	200	0
Fe88J1	Apigenin	0	100	0
Fe88J1	Apigenin	0	50	0
Fe88J1	Resveratrol	0	400	0
Fe88J1	Resveratrol	0	200	0
Fe88J1	Resveratrol	0	100	0
Fe88J1	Resveratrol	0	50	0
At71D1	Scopoletin	0	400	0
At71D1	Scopoletin	0	200	0
At71D1	Scopoletin	0	100	0
At71D1	Scopoletin	0	50	0
At71D1	Apigenin	0	400	0
At71D1	Apigenin	0	200	0
At71D1	Apigenin	0	100	0
At71D1	Apigenin	0	50	0
At71D1	Resveratrol	0	400	0
At71D1	Resveratrol	0	200	0
At71D1	Resveratrol	0	100	0
At71D1	Resveratrol	0	50	0
At71C1	Scopoletin	0	400	0
At71C1	Scopoletin	0	200	0
At71C1	Scopoletin	0	100	0
At71C1	Scopoletin	0	50	0
At71C1	Apigenin	0	400	0
At71C1	Apigenin	0	200	0
At71C1	Apigenin	0	100	0
At71C1	Apigenin	0	50	0
At71C1	Resveratrol	0	400	0
At71C1	Resveratrol	0	200	0
At71C1	Resveratrol	0	100	0
At71C1	Resveratrol	0	50	0

Supplementary Table 3. Initial rates of apigenin glucosylation according to the GT1 enzyme, its concentration, and apigenin concentration.

UGT/GT1 name	Enzyme concentration (mg/L)	[apigenin] (µM)	Observed rate (min^{-1})
***Zm*UGT708A6**	**20**	**50**	**8.8**
***Zm*UGT708A6**	**20**	**400**	**ND**
***Zm*UGT708A6**	**80**	**50**	**9.4**
***Zm*UGT708A6**	**80**	**400**	**ND**
***Fi*88A10**	**20**	**50**	**60**
***Fi*88A10**	**20**	**400**	**ND**
***Fi*88A10**	**80**	**50**	**20**
***Fi*88A10**	**80**	**400**	**ND**
***At*71C1**	**20**	**50**	**high**
***At*71C1**	**20**	**400**	**4200**
***At*71C1**	**80**	**50**	**high**
***At*71C1**	**80**	**400**	**2700**

ND: no reaction detected. high: >30% conversion observed in the first 0.5 min, initial rates could not be determined. Note that the observed rates relate to the exact conditions (293 K, HEPES 50 mM pH 7, indicated concentrations of enzyme and acceptor) and do not necessarily reflect the k_{cat} of the enzymes.

Bidart, G. N. *et al.* (2022) ‘Family 1 glycosyltransferase UGT706F8 from Zea mays selectively catalyzes the synthesis of silibinin 7-O-β-D- glucoside’, *ACS Sustainable Chemistry & Engineering*, in press(in press), pp. 1–24. Available at: 10.1021/acssuschemeng.1c07593.

Ferreyra, L. M. F. *et al.* (2013) ‘Identification of a Bifunctional Maize *C*- and O-Glucosyltransferase’, *Journal of Biological Chemistry*, 288(44), pp. 31678–31688. doi: 10.1074/jbc.M113.510040.

Liu, H. and Nidetzky, B. (2021) ‘Leloir glycosyltransferases enabled to flow synthesis : Continuous production of the natural *C*-glycoside nothofagin’, *Biotechnol Bioeng.*, 118(118), pp. 4402–4413. doi: 10.1002/bit.27908.

Modolo, L. V. *et al.* (2007) ‘A functional genomics approach to (iso) flavonoid glycosylation in the model legume *Medicago truncatula*’, *Plant. Mol. Biol.*, 64(64), pp. 499–518. doi: 10.1007/s11103-007-9167-6.

Teze, D. *et al.* (2021) ‘*O*-/*N*-/*S*-Specificity in glycosyltransferase catalysis: from mechanistic understanding to engineering’, *ACS Catalysis*, 11(11), pp. 1810–1815.

Wang, L. *et al.* (2013) ‘Comparing the acceptor promiscuity of a *Rosa hybrida* glucosyltransferase RhGT1 and an engineered microbial glucosyltransferase OleD^{PSA} toward a small flavonoid library’, *Carbohydrate research*, 368, pp. 73–77. doi: 10.1016/j.carres.2012.12.012.

Yang, M. *et al.* (2018) ‘Functional and informatics analysis enables glycosyltransferase activity prediction’, *Nature Chemical Biology*. Springer US, 14(12), pp. 1109–1117. Available at: http://dx.doi.org/10.1038/s41589-018-0154-9.
